# Supplementary material for: Long-term memory plasticity in a decade-long connectivity study post anterior temporal lobe resection
Source: Nat Commun. 2025 Jan 15;16:692. doi: 10.1038/s41467-024-55704-x (PMC11735635; doi:10.1038/s41467-024-55704-x)
Supplement: Supplementary file 1 — Supplementary Information [file 41467_2024_55704_MOESM1_ESM.pdf]

Supplementary Information

Long-Term Memory Plasticity in A Decade-Long Connectivity Study Post Anterior Temporal Lobe Resection

Marine Fleury*, Lawrence P. Binding, Peter Taylor, Fenglai Xiao*, Davide Giampiccolo, Sarah Buck, Gavin P. Winston, Pamela J. Thompson, Sallie Baxendale, Andrew W. McEvoy, Matthias J. Koepp, John S. Duncan, Meneka K. Sidhu

Supplementary Results

Subjects

The initial cohort for this longitudinal study consisted of 90 individuals with temporal lobe epilepsy (TLE), including 43 with left-sided TLE, and 29 control participants. Of the 90 TLE participants, 65 underwent anterior temporal lobectomy (ATLR), with 30 having left-sided surgery. All participants were invited to participate in long-term follow-up assessments, of which 25 postsurgical individuals and 10 controls returned for long-term study. Among the 40 participants who did not return (18 of whom had left-sided ATLR), clinical data was available for 35 individuals. Of these, 74% (26/35) were seizure-free with an ILAE outcome of 1 at the 12-month clinical follow-up, including 75% of the left ATLR group and 74% of the right ATLR group. However, these participants did not consent to participate in the long-term follow-up imaging studies, and as such, no additional outcomes are reported for those who chose not to take part.

Group differences in memory outcome

One-way ANOVAs were used on parametric memory and IQ z-scores and post-hoc tests were corrected using Tukey's Honestly Significant Difference (HSD) adjustment.

Preoperatively, controls had significantly higher IQ than presurgical left ATLR (verbal IQ: P = 0.003, 95% CI = [0.38; 1.97]; performance IQ: P = 0.010, 95% CI = [0.19; 1.58]), and right ATLR patients (verbal IQ: P = 0.003, 95% CI = [0.36; 1.92]; performance IQ: P = 0.008, 95% CI = [0.21; 1.58]).

Across preoperative and postoperative timepoints, there was no significant difference in verbal or visual memory z-scores between left and right ATLR groups, as shown below. All tests were corrected for multiple comparisons via Tukey's HSD adjustment.

Verbal memory. Before surgery: *P* = 0.22, 95% CI = [-0.35; 1.93]; 3-12 months: *P* = 0.404, 95% CI = [-0.62; 2.01]; 10 years: *P* = 0.84, 95% CI = [-1.04; 1.66].

Visual memory. Before surgery: *P* = 0.99, 95% CI = [-0.74; 0.84]; 3-12 months: *P* = 0.073, 95% CI = [-1.75; 0.064]; 10 years: *P* = 0.51, 95% CI = [-1.56; 0.59].

Impact of clinical factors:

From 3-12 months to 10 years post-ATLR, people who ceased medication recovered significantly better in visual memory function than those who continued ASMs, but not in verbal memory (visual: *Z* = 2.08; *P* = 0.036; verbal: *Z* = -0.87; *P* = 0.40). Post-hoc analyses showed significant ASM effect on visual memory after right but not left ATLR (right: *Z* = 2.37, *P* = 0.012; left: *Z* = 0.28, *P* = 0.79). Ongoing seizures 10 years post-surgery did not affect memory recovery (visual: *Z* = 1.44; *P* = 0.16; verbal: *Z* = 0.39; *P* = 0.71).

Individuals with HS preoperatively showed worse 10-year verbal and visual memory functions than those without HS pathology (verbal: $Z = -2.70$, $P = 0.0045$; visual: $Z = -2.11$, $P = 0.029$). Post-hoc analyses showed significant HS effect after left ATLR on verbal memory ($Z = -1.86$, $P = 0.045$) but not visual memory ($Z = -1.53$, $P = 0.14$) nor right ATLR (verbal: Z = -1.86, $P = 0.061$; visual: $Z = -1.87$, $P = 0.061$).

Impact of hippocampal sclerosis on long-term plasticity effects

Left ATLR: two samples t-test

Word remembered. People with HS compared to those without HS had reduced plasticity (i.e., less connectivity increases) from 3-12 months to 10 years between the remnant MTL seed and the bilateral posterior fusiform gyrus (remnant: MNI = -32 -58 -6, T=7.58; right: MNI=26 -76 -2, T=4.54). There was no difference in plasticity patterns from the contralesional hippocampus seed, nor any enhanced plasticity (more connectivity increases) between those with HS and those without HS.

Faces remembered. There was no difference from the remnant MTL seed between plasticity patterns of those with and without HS. People with HS had reduced plasticity between the contralesional hippocampus seed and the remnant posterior fusiform gyrus (MNI:-22 -48 -16, T=3.74).

Right ATLR: two samples t-test

Word remembered. No difference from the contralesional hippocampus seed. People with HS compared to those without HS had less plasticity (i.e., less longitudinal increases in

connectivity) between the remnant MTL seed and the remnant fusiform gyrus (MNI=34 -38 -14, T=2.96).

Faces remembered. People with HS compared to those without HS had more plasticity (i.e., greater increases in connectivity) from 3-12 months to 10 years between the contralesional hippocampus seed and the left posterior fusiform gyrus (MNI: -38 -46 -12, T=4.20), but reduced connectivity with the remnant posterior fusiform gyrus (MNI: 34 -46 -12, T=3.53). People with HS compared to those without HS had reduced plasticity (less connectivity increases) between the remnant MTL seed and the remnant posterior parahippocampus (MNI=36 -42 -8, T=3.52).

Shorter-term plasticity (3-12 months) supportive of longer-term improvement in postoperative memory

To further elucidate the neural mechanisms underlying long-term memory recovery, we investigated the changes in connectivity from before to shortly after ATLR that remain supportive of memory recovery over the long term (beyond 3-12 months in PWE compared to changes observed in healthy controls. Similar methods to the primary long-term plasticity analysis were employed, using separate mixed ANOVAs within a flexible factorial design for each MTL seed and memory task, with both IQ levels and change in ASM intake as confounding variables. Individual memory change (list and design learning) from 3-12 months to 10 years was treated as the regressor of interest.

Left ATLR relative to controls

Words remembered. Short-term after surgery compared to preoperatively, functional connectivity from the remnant MTL seed that was reduced with the remnant posterior fusiform

gyrus (MNI= -38 -46 -18, T=3.51) but was increased with the right middle cingulate cortex (MNI = 18 -32 48, T=3.40) and right superior parietal gyrus (MNI=22 -48 70, T=3.56) correlated with people who showed improvement in verbal memory over the long term. From the contralesional hippocampal seed, long-term verbal memory improvement correlated with increased 3-12-month connectivity with the right anterior and posterior OFC (anterior: MNI=22 38 -16, T=3.50, posterior: MNI=34 34 -14, T=4.28), right middle frontal (MNI=34 16 42, T=3.98) and bilateral parietal areas (left supramarginal: MNI=-62 -42 24, T=4.28; right superior parietal: MNI=30 -66 54, T=3.85) compared to before surgery. There were also adaptive decreases in right hippocampal connectivity with the right inferior frontal gyrus (MNI=40 16 14, T=3.68), right inferior OFC (MNI=36 30 -6, T=3.53) and right angular gyrus (MNI=44 -48 36, T=3.57).

Faces remembered. Short-term after surgery compared to preoperatively, people who had reduced functional connectivity from the remnant MTL seed with the right posterior fusiform gyrus (MNI=26 -66 -4, T=3.66) showed improvement in longer term visual memory. From the contralesional hippocampus seed, reduced 3-12-month connectivity with the right superior temporal gyrus (MNI=44 -36 14, T=3.57) but increased with the right middle temporal gyrus (MNI=58 -62 8, T=3.69) correlated with people who improved over the long term.

Right ATLR relative to controls

Words remembered. Short-term post-surgery compared to preoperatively, people who had connectivity from the contralesional left hippocampus that was reduced with the remnant posterior fusiform gyrus (MNI=30 -82 -6, T=3.48) and right parietal cortex (angular gyrus: MNI=56 -60 36, T=3.58) but increased with the left amygdala (MNI=-18 -4 16, T=4.95), right middle temporal gyrus (MNI=42 -42 -2, T=3.51) and right middle cingulate cortex (MNI=18 -40 30, T=3.40) showed improvement in longer term memory. From the remnant right MTL

seed, people with more functional connectivity with the right anterior OFC (MNI=38 36 -16, T=4.53) at 3-12-month compared to preoperatively showed improvement in long-term verbal memory.

Faces remembered. From the contralesional hippocampus seed, people who had increased functional connectivity with the right cuneus (MNI=20 -70 20, T=3.72) at 3-12months compared to before surgery also exhibited long-term visual memory improvement. From the remnant MTL seed, reduced connectivity with the left inferior OFC (MNI=-44 32 -12, T=3.51) and (MNI=22 -70 16, T=4.11) and right basal ganglia (external globus pallidus: MNI= 14 2 -8, T=3.82) correlated with people with improvement in long-term visual memory.

Sensitivity analysis: Twelve months to 10 years plasticity

Current literature suggests that PWE may undergo distinct structural and functional reorganizations at 3 months compared to 12 months after surgery. The shorter-term follow-up (T2) consisted of a combination of both 12-month and 3-month timepoints for subjects who did not return at 12 months. To assess the impact on the robustness of long-term plasticity findings (T2 to T3 (10 years)), we compared longitudinal connectivity changes in the mixed 3-12-month follow-up cohort with those in a 12-month only cohort.

For each subject, we subtracted the preprocessed, normalised contrast image[1] of successful memory connectivity from the subject-level PPI analysis at T3 from the contrast image at T2 and vice versa, using the SPM toolbox ‘ImCalc’. This yielded two ‘connectivity difference’ contrast images per subject, MTL seed, and memory task: increased connectivity and decreased connectivity at T3 compared to T2 (3 or 12 months, depending on the subject) over the whole brain. At the second level of the random effects analysis, Two-sample t-tests then compared these functional connectivity changes between the ‘3-12-month’ and ‘12-month only’ groups,

separately for each ‘connectivity difference’ *T*-contrast, memory task, MTL seed, and ATLR group.

In left ATLR, this included 12 subjects in the combined ‘3-12-months’ cohort compared to eight in the ’12-month only’ group, while in right ATLR, there were 13 subjects in the combined cohort and 10 in the ’12-month only’ group. At an exploratory threshold $P < 0.05$, uncorrected, there were no significant differences in longitudinal connectivity changes (no suprathreshold difference) between the ‘3-12-month’ and the ‘12-month only’ cohorts during successful encoding of either words or faces for each ‘connectivity difference’ *T*-contrast, seeding from either MTL in any ATLR group. This is possibly due to the low number of 3-month compared to 12-month participants. Due to the lack of difference in connectivity changes, these groups were combined into a short-term postoperative group.

Language-disrupting antiseizure medication

Topiramate, Zonisamide, Carbamazepine and Lamotrigine are ASMs known to affect the functional language network and impact language function[2-5]. These medications may disproportionately impact verbal processes, thereby hindering verbal memory recovery. In our cohort of 25 individuals assessed for the effects of ASM on cognitive function, 15 remained on ASMs at the 10-year follow-up (8 following left ATLR). Of these, 10 were prescribed combinations of ASMs that included one or more of the language-disrupting ASMs, potentially contributing to the observed discrepancy between verbal and visual memory recovery in our cohort. Specifically, among the 15 people who continued medication:

Left ATLR group (n=8): six individuals were taking Topiramate, Zonisamide, Carbamazepine, or Lamotrigine. Of these, three were on polytherapy of two or more ASMs,

which is associated with greater disruption of cognitive function[6]. The remaining two individuals were either on Valproate or Levetiracetam monotherapy.

Right ATLR group (n=7): four individuals were taking either one of Topiramate, Zonisamide, Carbamazepine, or Lamotrigine. The remaining three persons were either on Valproate, Levetiracetam, or Oxcarbazepine. All 7 individuals in the right ATLR group were on monotherapy.

Additional descriptive statistics were conducted, and suggested that verbal memory recovery was more disrupted in individuals taking ASMs affecting verbal processes:

- Individuals taking language-disrupting ASMs (n = 10): median verbal memory change = **0** (IQR = -0.45–0.70).
- Individuals taking other ASMs (n=5): median verbal memory change = **0.63** (IQR= 0–1.36), a clinically significant improvement above the upper limit of the RCI (RCI-improvement of 0.31) using a 95% confidence interval.

These descriptive statistics suggest that verbal memory recovery may be sensitive to ASMs that disrupt language-related functions.

Supplementary Methods

Subjects

All subjects included in this study returned for the 10-year neuropsychometry and fMRI assessments. Supplementary table 1 below outlines the number people at each follow-up.

Table S1 Number of healthy controls and people who had anterior temporal lobe resection at each timepoint.

	Baseline	3 months	12 months	10 years
**Controls**	10	9	7	10
**Left resection**	12	12	8	12
**Right resection**	13	13	10	13

The number of individuals who had both neuropsychometry and neuroimaging assessments done at each experimental timepoint is outlined. This includes the preoperative assessment, and 3-month, 12-month and 10-year follow-ups after people underwent left- or right-sided resection, and the equivalent first and follow-up tests in healthy matched controls.

Neuropsychological tests

Standardized neuropsychometry was administered at equivalent time-points in patients and controls; before, at median 3-month and 12-month after surgery, and up to 10 years postoperatively (median 9 years).

Assessment of intellectual functioning was performed using Full-Scale IQ (FSIQ) of the Wechsler Adult Intelligence Scale (WAIS)[7] in controls, and with the National Adult Reading Test (NART-2)[8-10] for metrics of premorbid verbal and performance IQ in patients.[11,12] NART-IQ is reported to give the most reliable and precise estimates of patients' FSIQ.[13]

The verbal learning BMIPB-I and II did not differ between versions. Over five trials, participants learned and recalled a list of 15 words read aloud (i.e., maximum score of 75). Design learning required participants to learn over five trials a design presented for 10 seconds and composed of nine (10 for BMIPB-II) connected lines on a 4 x 4 dot matrix. Following each presentation, the participant is asked to reproduce the design from memory on a blank grid. For

the BMIPB-I, the total number of correctly connected dots is recorded, generating a maximum score of 45 (50 for BMIPB-II).

The conversion of memory scores into z-scores, using ageing norms of corresponding BMIPB version, accounted for change in BMIPB versions and age-related differences.[14] Memory change represented the difference between z-scores of short-term and long-term follow-ups. Improvement or decline were considered clinically significant based on reliable change index (RCI) upper and lower limits, using 95% confidence interval (CI), as described in neuropsychological and imaging studies.[11,14-16] For verbal memory, this meant an RCI improvement of ≥ 0.31 and a RCI decline of ≥ 0.40. For visual memory, an RCI improvement of ≥ 0.58 and RCI decline of ≥ 0.16 were deemed clinically significant. The RCI probes meaningful change by adjusting for test reliability and practice effect in a test-retest context.[14]

Magnetic resonance data acquisition

Preoperatively and at the short-term follow-up, participants were scanned on a 3T GE Signa Excite HDx MRI scanner, with a 20-channel head coil[17]. An axial 3D T1-weighted sequence (FSPGR) was acquired[11]. For the memory fMRI, T2*-weighted gradient echo planar images (EPI) were acquired using 36 contiguous oblique axial slices per volume, 24-cm field of view, 2.5 mm slice thickness (0.3 mm gap), 96 x 96 matrix interpolated to 128 x 128 during image reconstruction, 2.5 in-plane resolution, and 2.5 SENSE factor (TE/TR = 25/27500 ms).[18,19]

At the long-term follow-up, data was acquired on a 3T GE Discovery MR750, with a 32-channel head coil[17]. An axial 3D T1-weighted sequence (FSPGR) was acquired[20]. Memory fMRI gradient-echo planar T2*-weighted fMRI was acquired using 50 contiguous oblique axial

slices, 24-cm field of view, 2.4 mm slice thickness (0.1 mm gap), 64 × 64 matrix, 3.75 in-plane resolution, and 2.0 SENSE factor (TE/TR = 22/27500 ms).

At each scanning time-point, the field of view covered the temporal and frontal lobes, and slices were aligned on the sagittal view with the long axis of the hippocampus.[21]

Functional memory paradigm

The material-specific memory fMRI paradigm consisted of black-and-white faces and words visually presented on a magnetic resonance compatible screen and viewed through a mirror during a single scanning session at each time-point.[18,19,21,22] Participants were instructed to memorize a total of 100 faces (combination of unfamous neutral and fearful photographs), and 100 single concrete nouns with or without emotional valence. Using a button-box, participants were asked to make a subjective decision about the pleasantness of the presented item in order to foster deep encoding.[23] Visual and verbal items were presented for 3s in blocks with a total of 10 blocks. One block contained 10 faces (five fearful) and 10 words (two emotionally averse) and was followed by crosshair fixation. Jitter and random sampling were introduced through a 3s interstimulus interval (*vs*. 2.75s TR).[19]

Forty minutes after scanning, participants performed words and faces recognition tests, separately. The same 100 stimuli were randomly presented intermixed at the same speed as displayed inside the scanner, with an additional 50 novel faces/words as foils. Participants were asked to classify items as remembered, familiar (if unsure), or novel using a button-box. Each stimulus encoded in the scanner was subsequently categorized as successfully remembered, familiar or forgotten. At each scanning timepoint, an identical memory fMRI paradigm was performed with different words and faces.

Data analysis

Generation of event-related contrasts

Event-related spmT maps of subsequent memory (remembered, familiar, forgotten) were generated for each subject and separately for words or faces on SPM12 via random-effects analysis.[21,24]

At the subject-level, activation patterns at encoding of stimuli that were subsequently remembered, familiar, or forgotten were modelled in a general linear model (GLM) of a blocked design. The delta function of each event-related condition was convolved with the canonical hemodynamic response function (HRF) and its temporal derivative. Six regressors of interest were created; words and faces subsequently remembered, familiar, or forgotten. Six motion parameters were added as confounds. For each subject, the resulting event-related statistical map was used for single-level gPPI connectivity analysis.

Event-related functional connectivity analysis

Psychophysiological interaction[25,26] analysis allows the investigation of task-modulated functional couplings between a seed and whole-brain regions. A generalized form of PPI was used to span the entire experimental space,[27] wherein beta-estimates of all six event-related subsequent memory conditions were modelled. For each participant, ROI to voxelwise whole-brain analysis was performed based on a MATLAB (R2020b) script template from McLaren et al.,[27] that was adapted to work within the SPM12 framework.

Using each participant's event-related statistical map, each subject-level gPPI model included three regressors: the time-courses of event-related task conditions (subsequent verbal and

visual memory), time-series of one MTL seed, and of the PPI term (i.e., task*seed interaction). All six event-related task conditions were modelled to better probe the specific effect of successful subsequent verbal and visual memory.[27] Within each anatomical MTL seed, the average seed's time-course was extracted, over the eigen variable to avoid skewing the contribution of distinct voxels in different subjects.

The seed regressor identifies voxels that correlate with the MTL seed in general. Task*seed interaction term (i.e., PPI term) was formed by deconvolving the HRF from each MTL seed's time-course, multiplying the deconvolved output by each task condition's time-series separately, and finally re-convolving the time-series with the HRF.[27] Deriving the PPI term from HRF deconvolution allows to model connectomics dynamics at the neural level.[28] Subject-level resulting statistical parametric map presented whole-brain activations that were significantly more correlated with the seed during a specific event-related task than during the other conditions, based on the PPI term prediction and with physiologic and psychologic variables treated as nuisance regressors. For the study's scope, t-contrasts of areas functionally coupled to the MTL seed during encoding of either words or faces subsequently remembered were generated.

Thus, for each participant, a separate GLM was performed for each MTL seed at the early and late postoperative timepoints. The exact same conditions' time-series and MTL seeds were used across subjects/within groups, and postoperative time-points. We ran the gPPI using left and right hippocampi as MTL seed regions in healthy participants, and in patients who had ATLR using the contralateral hippocampus, and remnant hippocampus and parahippocampal gyrus. Single-level gPPI t-contrasts of successful subsequent memory were applied to group-level random-effects analyses for functional connectivity estimation and comparison across

groups. Group analyses are described in the main manuscript's Methods – longitudinal assessment of the functional memory network.

Eigenvariates extraction for graphical presentation

Based on full factorial ANOVAs of 'connectivity difference' contrast images[1], eigenvariates were extracted from the functional connectivity changes in ATLR compared with healthy controls, controlling for change in ASM intake and IQ. These eigenvariates, adjusted for the F-contrast of interest, were used to predict individual memory changes, thereby confirming the gPPI construct validity in modelling neural substrates of memory function.

Supplementary References

1 Bonelli, S. B. *et al.* Memory reorganization following anterior temporal lobe resection: a longitudinal functional MRI study. *Brain* **136**, 1889-1900 (2013). https://doi.org/10.1093/brain/awt105

2 Yasuda, C. L. *et al.* The effect of topiramate on cognitive fMRI. *Epilepsy Res* **105**, 250-255 (2013). https://doi.org/10.1016/j.eplepsyres.2012.12.007

3 Xiao, F. *et al.* Effect of anti-seizure medications on functional anatomy of language: a perspective from language functional magnetic resonance imaging. *Frontiers in neuroscience* **15**, 787272 (2022).

4 Wandschneider, B. *et al.* Effect of topiramate and zonisamide on fMRI cognitive networks. *Neurology* **88**, 1165-1171 (2017). https://doi.org/10.1212/wnl.0000000000003736

5 Xiao, F. *et al.* Effects of carbamazepine and lamotrigine on functional magnetic resonance imaging cognitive networks. *Epilepsia* **59**, 1362-1371 (2018). https://doi.org/10.1111/epi.14448

6 Witt, J.-A., Elger, C. E. & Helmstaedter, C. Adverse cognitive effects of antiepileptic pharmacotherapy: Each additional drug matters. *European Neuropsychopharmacology* **25**, 1954-1959 (2015). https://doi.org/https://doi.org/10.1016/j.euroneuro.2015.07.027

7 Wechsler, D. *WAIS-III: Administration and scoring manual: Wechsler adult intelligence scale*. (Psychological Corporation, 1997).

8 Nelson, H. E. & O'Connell, A. Dementia: The Estimation of Premorbid Intelligence Levels Using the New Adult Reading Test. *Cortex* **14**, 234-244 (1978). https://doi.org/https://doi.org/10.1016/S0010-9452(78)80049-5

9 Nelson, H. & Willison, J. National adult reading test (NART) manual. *Windsor, Bershire, UK: NFER-Nelson* (1982).

10 Nelson, H. & Willison, J. The revised national adult reading test–test manual. *Windsor, UK: NFER-Nelson* **991**, 1-6 (1991).

11 Sone, D. *et al.* Optimal Surgical Extent for Memory and Seizure Outcome in Temporal Lobe Epilepsy. *Annals of Neurology* **91**, 131-144 (2022). https://doi.org/https://doi.org/10.1002/ana.26266

12 Caciagli, L. *et al.* The cognitive phenotype of juvenile absence epilepsy and its heritability: An investigation of patients and unaffected siblings. *medRxiv*, 2022.2004. 2012.22273461 (2022).

13 Bright, P., Jaldow, E. & Kopelman, M. The National Adult Reading Test as a measure of premorbid intelligence: A comparison with estimates derived from demographic variables. *Journal of the International Neuropsychological Society* **8**, 847-854 (2002). https://doi.org/10.1017/S1355617702860131

14 Baxendale, S. & Thompson, P. The association of cognitive phenotypes with postoperative outcomes after epilepsy surgery in patients with temporal lobe epilepsy. *Epilepsy & Behavior* **112**, 107386 (2020). https://doi.org/https://doi.org/10.1016/j.yebeh.2020.107386

15 Baxendale, S., Thompson, P., Harkness, W. & Duncan, J. Predicting Memory Decline Following Epilepsy Surgery: A Multivariate Approach. *Epilepsia* **47**, 1887-1894 (2006). https://doi.org/https://doi.org/10.1111/j.1528-1167.2006.00810.x

16 Binding, L. P. *et al.* Contribution of White Matter Fiber Bundle Damage to Language Change After Surgery for Temporal Lobe Epilepsy. *Neurology* **100**, e1621-e1633 (2023). https://doi.org/10.1212/wnl.0000000000206862

17 Taylor, P. N. *et al.* The impact of epilepsy surgery on the structural connectome and its relation to outcome. *NeuroImage: Clinical* **18**, 202-214 (2018). https://doi.org/https://doi.org/10.1016/j.nicl.2018.01.028

18 Bonelli, S. B. *et al.* Imaging memory in temporal lobe epilepsy: predicting the effects of temporal lobe resection. *Brain* **133**, 1186-1199 (2010). https://doi.org/10.1093/brain/awq006

19 Sidhu, M. K. *et al.* A functional magnetic resonance imaging study mapping the episodic memory encoding network in temporal lobe epilepsy. *Brain* **136**, 1868-1888 (2013). https://doi.org/10.1093/brain/awt099

20 Vos, S. B. *et al.* Hippocampal profiling: Localized magnetic resonance imaging volumetry and T2 relaxometry for hippocampal sclerosis. *Epilepsia* **61**, 297-309 (2020). https://doi.org/10.1111/epi.16416

21 Sidhu, M. K. *et al.* Memory network plasticity after temporal lobe resection: a longitudinal functional imaging study. *Brain* **139**, 415-430 (2016). https://doi.org/10.1093/brain/awv365

22 Fleury, M. *et al.* Episodic memory network connectivity in temporal lobe epilepsy. *Epilepsia* **63**, 2597-2622 (2022). https://doi.org/https://doi.org/10.1111/epi.17370

23 Craik, F. I. Levels of processing: past, present. and future? *Memory* **10**, 305-318 (2002). https://doi.org/10.1080/09658210244000135

24 Monti, M. M. Statistical Analysis of fMRI Time-Series: A Critical Review of the GLM Approach. *Front Hum Neurosci* **5**, 28 (2011). https://doi.org/10.3389/fnhum.2011.00028

25 Friston, K. J. *et al.* Psychophysiological and Modulatory Interactions in Neuroimaging. *NeuroImage* **6**, 218-229 (1997). https://doi.org/https://doi.org/10.1006/nimg.1997.0291

26 O'Reilly, J. X., Woolrich, M. W., Behrens, T. E. J., Smith, S. M. & Johansen-Berg, H. Tools of the trade: psychophysiological interactions and functional connectivity. *Social cognitive and affective neuroscience* **7**, 604-609 (2012). https://doi.org/10.1093/scan/nss055

27 McLaren, D. G., Ries, M. L., Xu, G. & Johnson, S. C. A generalized form of context-dependent psychophysiological interactions (gPPI): A comparison to standard approaches. *NeuroImage* **61**, 1277-1286 (2012). https://doi.org/https://doi.org/10.1016/j.neuroimage.2012.03.068

28 Gitelman, D. R., Penny, W. D., Ashburner, J. & Friston, K. J. Modeling regional and psychophysiologic interactions in fMRI: the importance of hemodynamic deconvolution. *NeuroImage* **19**, 200-207 (2003). https://doi.org/https://doi.org/10.1016/S1053-8119(03)00058-2
