# Supplementary material for: Long-term memory plasticity in a decade-long connectivity study post anterior temporal lobe resection
Source: Nat Commun. 2025 Jan 15;16:692. doi: 10.1038/s41467-024-55704-x (PMC11735635; doi:10.1038/s41467-024-55704-x)
Supplement: Supplementary file 2 — Reporting Summary [file 41467_2024_55704_MOESM2_ESM.pdf]

nature portfolio

Corresponding author(s): Marine N. Fleury
Fenglai Xiao

Last updated by author(s): Nov 21, 2024

Reporting Summary

Nature Portfolio wishes to improve the reproducibility of the work that we publish. This form provides structure for consistency and transparency in reporting. For further information on Nature Portfolio policies, see our Editorial Policies and the Editorial Policy Checklist.

Statistics

For all statistical analyses, confirm that the following items are present in the figure legend, table legend, main text, or Methods section.

n/a	Confirmed	
☐	☒	The exact sample size (*n*) for each experimental group/condition, given as a discrete number and unit of measurement
☐	☒	A statement on whether measurements were taken from distinct samples or whether the same sample was measured repeatedly
☐	☒	The statistical test(s) used AND whether they are one- or two-sided<br>*Only common tests should be described solely by name; describe more complex techniques in the Methods section.*
☐	☒	A description of all covariates tested
☐	☒	A description of any assumptions or corrections, such as tests of normality and adjustment for multiple comparisons
☐	☒	A full description of the statistical parameters including central tendency (e.g. means) or other basic estimates (e.g. regression coefficient) AND variation (e.g. standard deviation) or associated estimates of uncertainty (e.g. confidence intervals)
☐	☒	For null hypothesis testing, the test statistic (e.g. *F*, *t*, *r*) with confidence intervals, effect sizes, degrees of freedom and *P* value noted<br>*Give P values as exact values whenever suitable.*
☒	☐	For Bayesian analysis, information on the choice of priors and Markov chain Monte Carlo settings
☒	☐	For hierarchical and complex designs, identification of the appropriate level for tests and full reporting of outcomes
☒	☐	Estimates of effect sizes (e.g. Cohen's *d*, Pearson's *r*), indicating how they were calculated

Our web collection on statistics for biologists contains articles on many of the points above.

Software and code

Policy information about availability of computer code

Data collection	We applied the same material-specific memory fMRI paradigm as preoperatively (Sidhu et al., 2013. Brain; Fleury et al., 2022, Epilepsia).<br><br>The material-specific memory fMRI paradigm consisted of black-and-white faces and words visually presented on a magnetic resonance compatible screen and viewed through a mirror during a single scanning session at each time-point. Participants were instructed to memorize a total of 100 faces (combination of unfamous neutral and fearful photographs), and 100 single concrete nouns with or without emotional valence. Using a button-box, participants were asked to make a subjective decision about the pleasantness of the presented item in order to foster deep encoding.23 Visual and verbal items were presented for 3s in blocks with a total of 10 blocks. One block contained 10 faces (five fearful) and 10 words (two emotionally averse) and was followed by crosshair fixation. Jitter and random sampling were introduced through a 3s interstimulus interval (vs. 2.75s TR).
Data analysis	Subject level statistical T-maps were generated on Statistical Parametric Mapping 12 (SPM12; Wellcome Department of Cognitive Neurology, Institute of Neurology, London, UK; http:// www.fil.ion.ucl.ac.uk/spm/) and used for generalized psychophysiological interaction (PPI) analysis. Generalized PPI was done by implementing a publicly available toolbox (McLaren, Ries & Johnson 2012. Neuroimage).<br>All imaging data was analysed on Statistical Parametric Mapping 12 (SPM12; Wellcome Department of Cognitive Neurology, Institute of Neurology, London, UK, http:// www.fil.ion.ucl.ac.uk/spm/) and clinical and neuropsychology data on R 4.0.5.

For manuscripts utilizing custom algorithms or software that are central to the research but not yet described in published literature, software must be made available to editors and reviewers. We strongly encourage code deposition in a community repository (e.g. GitHub). See the Nature Portfolio guidelines for submitting code & software for further information.

Data

Policy information about availability of data

All manuscripts must include a data availability statement. This statement should provide the following information, where applicable:

- Accession codes, unique identifiers, or web links for publicly available datasets
- A description of any restrictions on data availability
- For clinical datasets or third party data, please ensure that the statement adheres to our policy

Data availability: Source data are provided with this paper and can be accessed at https://github.com/marine-fleury/memory-plasticity.git.
Raw data used for boxplots and scatter plots are provided in a single Excel file with specific data used for different analyses in separate sheets. Data used for analysis and make figures is made publicly available on Github. https://github.com/marine-fleury/memory-plasticity.git

Research involving human participants, their data, or biological material

Policy information about studies with human participants or human data. See also policy information about sex, gender (identity/presentation), and sexual orientation and race, ethnicity and racism.

Reporting on sex and gender

Sex was considered in the study design and during data collection to balance acquired data in evenly matched patient groups. Healthy controls were sex matched.
Sex was determined based on self-report and/or electronic health records.
Disaggregated numbers for individual experiments is provided in the source data.
Two-sided Fisher exact test showed a non-significant difference in sex between individuals undergoing epilepsy surgery (ATLR) and control groups. Each group was comparable in sex proportion: in left ATLR there were 7 males and 5 females, in right ATLR there were 4 males and 9 females, and in controls there were 4 males and 5 females. Every participants had consented to their sex being recorded. We did not perform further analysis based on participants' sex as they were comparable between groups, and we were interested in the individual changes in memory network connectivity in relation to individual memory recovery in post-surgical people with epilepsy, controlling for factors like aging via the control group.

Reporting on race, ethnicity, or other socially relevant groupings

N/a

Population characteristics

We studied 25 individuals with medically refractory Temporal Lobe Epilepsy undergoing standard unilateral Anterior Temporal Lobe Resection (ATLR) from 2009 to 20121 at the National Hospital for Neurology and Neurosurgery (NHNN), London, United Kingdom. All patients showed ipsilateral seizure onset to the temporal lobe and underwent standard en bloc temporal lobe resection of the hippocampus with a posterior resection margin at the mid-brainstem level. There were 12 left-sided ATLR (seven males, median preoperative age 38 years, interquartile range (IQR) 28-41) and 13 right-sided (four males, median preoperative age 38 years, IQR 29-50).

Ten healthy, English-proficient, matched-controls (four males, aged 27-50) were assessed at similar intervals. Controls and left/right ATLR groups were comparable for language dominance, handedness, sex, and age.

Preoperative and postoperative seizure frequency were collected from seizure diaries; it consisted of the average total number of focal impaired awareness seizures per month and focal to bilateral tonic–clonic seizures.

Recruitment

All participants were identified from previous surgical database, in which participants who completed the same neuropsychology structural MRI, and memory fMRI assessments across study timepoints were selected. Neuropsychology and neuroimaging data was acquired at four timepoints: preoperatively, and at a median 3-month (IQR = 3-4) and 12-month (IQR = 11-13.5) post-surgery, and up to 10 years postoperatively (median = 9, IQR = 8-10). Eight left-sided and 10 right-sided ATLR cases completed assessments at all postoperative follow-ups.

A potential bias of longitudinal design is that, although we included all participants who were keen to return for a further assessment, the majority of people were seizure free long-term after surgery. Consequently, our long-term assessment data may predominantly reflect successful surgical cases. To mitigate this bias and gain a more comprehensive understanding, future studies could include neuropsychology and imaging follow-ups with individuals who continue to experience recurrent seizures. Such follow-ups could provide valuable insights into the mechanisms underlying their rehabilitation and inform more nuanced interpretations of our results. This issue for generalisation has been discussed in the limitations of the main manuscript.

Ethics oversight

All participants provided written informed consent in accordance with the Declaration of Helsinki. The NHNN and Institute of Neurology Joint Research Ethics Committee approved this research (18/LO/1447).

Note that full information on the approval of the study protocol must also be provided in the manuscript.

Field-specific reporting

Please select the one below that is the best fit for your research. If you are not sure, read the appropriate sections before making your selection.

[x] Life sciences   [ ] Behavioural & social sciences   [ ] Ecological, evolutionary & environmental sciences

Life sciences study design

All studies must disclose on these points even when the disclosure is negative.

Sample size	The sample size for our study was determined by the number of participants who agreed to return for long-term follow-up 10 years post-ATLR. A longitudinal fMRI approach maximizes the power to detect time-related changes and to separate effects to the within- versus between-person level, assuming that the variability between subjects is significantly greater than the variability between sessions for a given participant (McCormick, 2023; Skup, 2010).<br><br>Previous fMRI studies with similar numbers and design to ours have shown significant fMRI activations (McCormick et al. 2017; Qu et al. 2015; Pfeifer et al. 2013). We corroborate this by showing group activations that survive stringent multiple comparisons in regions of a-priori interest and significant activations despite a specific flexible factorial model. To validate our findings, future studies with larger sample sizes should be conducted.<br><br>References:<br>McCormick, Ethan M., et al. "The hitchhiker's guide to longitudinal models: A primer on model selection for repeated-measures methods." Developmental cognitive neuroscience 63 (2023): 101281.<br>Skup, Martha. "Longitudinal fMRI analysis: A review of methods." Statistics and its interface 3.2 (2010): 232.<br>McCormick, Ethan M., Yang Qu, and Eva H. Telzer. "Activation in context: differential conclusions drawn from cross-sectional and longitudinal analyses of adolescents' cognitive control-related neural activity." Frontiers in human neuroscience 11 (2017): 141.<br>Qu, Yang, et al. "Longitudinal changes in prefrontal cortex activation underlie declines in adolescent risk taking." Journal of Neuroscience 35.32 (2015): 11308-11314.<br>Pfeifer, Jennifer H., et al. "Longitudinal change in the neural bases of adolescent social self-evaluations: effects of age and pubertal development." Journal of Neuroscience 33.17 (2013): 7415-7419.
Data exclusions	Exclusion criteria included contraindication to MRI, non-proficient English speaker, and intelligence quotient (IQ) < 70.
Replication	The reproducibility of our experimental findings was verified through several measures. Post-operative image registration was conducted to ensure optimal data quality. This included field-bias correction of T1 images and normalization to a scanner-specific MNI template, realignment and slice-timing correction of imaging time-series, and normalization to an MNI template using a deep-learning registration method (EasyReg), which also performs EPI distortion correction and accounts for brain sag and mitigates distortions around the resection cavity (Iglesias. 2023. 'A ready-to-use machine learning tool for symmetric multi-modality registration of brain MRI'. Scientific Reports).<br><br>All data analyses were scripted, either on R-studio or on MATLAB, in order to increase the reproducibility and transparency of the analyses conducted. All reported connectivity within remnant medial temporal areas was validated against artifacts using exclusive MTL group-resection masks. Additionally, generalized PPI, compared to other connectivity tools such as standard PPI, models the entire experimental span, allowing the analysis of neural correlates highly specific to subsequent memory effects and better controls for both type I and II errors (McLaren, Ries & Johnson. 2012. Neuroimage).<br><br>The neuroimaging and behavioural analysis was conducted independently on data from all participants (n = 35 including 10 healthy controls) with each scan analysed separately at each of the time- points. Standard quality control measures were applied to ensure the accuracy and reliability of the results. Additionally, statistical analyses were performed using established protocols, and the sensitivity and supplementary post-hoc analyses performed as part of reviewers comments validated and strengthened the study findings.<br>To our knowledge this is the first description of longer term plasticity after epilepsy surgery hence there are no replications at this time point. This study design however was used to address dynamic changes in the memory network up to 12 months after epilepsy surgery. These results were peer reviewed and published in Brain 2016.<br><br>Reference:<br>Sidhu MK, Stretton J, Winston GP, McEvoy AW, Symms M, Thompson PJ, Koepp MJ, Duncan JS. Memory network plasticity after temporal lobe resection: a longitudinal functional imaging study. Brain. 2016 Feb;139(Pt 2):415-30. doi: 10.1093/brain/awv365. Epub 2016 Jan 10. PMID: 26754787; PMCID: PMC4805088.
Randomization	The side of anterior temporal lobe resection was the group allocation determinant for each surgical candidate with unilateral temporal lobe epilepsy. Healthy individuals were recruited to match sex and age of people with epilepsy and had no prior history of neurological condition.
Blinding	Blinding was not relevant in the study: we assessed the changes in the memory connectome following unilateral epilepsy surgery. Therefore, the resection side was the determinant of which group each participant was assigned to. Healthy individuals were sex and age matched.

Reporting for specific materials, systems and methods

We require information from authors about some types of materials, experimental systems and methods used in many studies. Here, indicate whether each material, system or method listed is relevant to your study. If you are not sure if a list item applies to your research, read the appropriate section before selecting a response.

Materials & experimental systems

n/a	Involved in the study
☐	☐ Antibodies
☐	☐ Eukaryotic cell lines
☐	☐ Palaeontology and archaeology
☐	☐ Animals and other organisms
☐	☒ Clinical data
☐	☐ Dual use research of concern
☐	☐ Plants

Methods

n/a	Involved in the study
☐	☐ ChIP-seq
☐	☐ Flow cytometry
☐	☒ MRI-based neuroimaging

Antibodies

Antibodies used	N/a
Validation	N/a

Eukaryotic cell lines

Policy information about cell lines and Sex and Gender in Research

Cell line source(s)	N/a
Authentication	N/a
Mycoplasma contamination	N/a
Commonly misidentified lines (See ICLAC register)	N/a

Palaeontology and Archaeology

Specimen provenance	N/a
Specimen deposition	N/a
Dating methods	N/a

☐ Tick this box to confirm that the raw and calibrated dates are available in the paper or in Supplementary Information.

Ethics oversight	N/a

Note that full information on the approval of the study protocol must also be provided in the manuscript.

Animals and other research organisms

Policy information about studies involving animals; ARRIVE guidelines recommended for reporting animal research, and Sex and Gender in Research

Laboratory animals	N/a
Wild animals	N/a
Reporting on sex	N/a
Field-collected samples	N/a
Ethics oversight	N/a

Note that full information on the approval of the study protocol must also be provided in the manuscript.

Clinical data

Policy information about clinical studies

All manuscripts should comply with the ICMJE guidelines for publication of clinical research and a completed CONSORT checklist must be included with all submissions.

Clinical trial registration	N/a
Study protocol	Described in Methods.
Data collection	Data was collected at the Chalfont Centre for Epilepsy (Epilepsy Society), Buckinghamshire, UK. Neuropsychology assessment, as well as structural and memory functional MRI were acquired at four timepoints: preoperatively, and at a median 3-month (IQR = 3-4) and 12-month (IQR = 11-13.5) post-surgery, and up to 10 years postoperatively (median = 9, IQR = 8-10). Eight left-sided and 10 right-sided ATLR cases completed assessments at all postoperative follow-ups.<br><br>To optimize acquired data and analysis, a 'short-term' assessment timepoint was introduced. It encompassed all data collected at 12 months, alongside data at 3 months from patients unable to attend 12-month follow-up (four left-sided and three right-sided). Throughout this manuscript, 'short-term' data refers to assessments conducted during the 3–12-month follow-up period (median = 11 months, IQR = 8-12), occurring from 2009 to 2013. Conversely, 'long-term' data pertains to the 10-year follow-up, conducted between 2019–2022.
Outcomes	Primary outcome: change in memory networks underlying cognitive recovery. Three-way ANCOVAs were conducted for each MTL seed and each successful memory contrast (words/faces), to investigate which differences in functional connectivity from 3–12-month and 10-year follow-ups were related with improvement in memory functions over this timeline.<br><br>Secondary Outcome: memory recovery (change in memory scores).<br>Memory scores were standardized into z-scores, using ageing norms of corresponding BMIPB version, accounting for version change and age-related differences. Memory change represented the difference between z-scores of short-term and long-term follow-ups. Improvement or decline were considered clinically significant based on reliable change index (RCI) upper and lower limits, using 95% confidence interval, as described in neuropsychological and imaging studies. RCI probes meaningful change by adjusting for test reliability and practice effect in a test-retest context.

Dual use research of concern

Policy information about dual use research of concern

Hazards

Could the accidental, deliberate or reckless misuse of agents or technologies generated in the work, or the application of information presented in the manuscript, pose a threat to:

No	Yes	
☒	☐	Public health
☒	☐	National security
☒	☐	Crops and/or livestock
☒	☐	Ecosystems
☒	☐	Any other significant area

Experiments of concern

Does the work involve any of these experiments of concern:

No	Yes	
☒	☐	Demonstrate how to render a vaccine ineffective
☒	☐	Confer resistance to therapeutically useful antibiotics or antiviral agents
☒	☐	Enhance the virulence of a pathogen or render a nonpathogen virulent
☒	☐	Increase transmissibility of a pathogen
☒	☐	Alter the host range of a pathogen
☒	☐	Enable evasion of diagnostic/detection modalities
☒	☐	Enable the weaponization of a biological agent or toxin
☒	☐	Any other potentially harmful combination of experiments and agents

Plants

Seed stocks	N/a
Novel plant genotypes	N/a
Authentication	N/a

ChIP-seq

Data deposition

☐ Confirm that both raw and final processed data have been deposited in a public database such as GEO.

☐ Confirm that you have deposited or provided access to graph files (e.g. BED files) for the called peaks.

Data access links<br>*May remain private before publication.*	N/a
Files in database submission	N/a
Genome browser session<br>(e.g. UCSC)	N/a

Methodology

Replicates	N/a
Sequencing depth	N/a
Antibodies	N/a
Peak calling parameters	N/a
Data quality	N/a
Software	N/a

Flow Cytometry

Plots

Confirm that:

☐ The axis labels state the marker and fluorochrome used (e.g. CD4-FITC).

☐ The axis scales are clearly visible. Include numbers along axes only for bottom left plot of group (a 'group' is an analysis of identical markers).

☐ All plots are contour plots with outliers or pseudocolor plots.

☐ A numerical value for number of cells or percentage (with statistics) is provided.

Methodology

Sample preparation	N/a
Instrument	N/a
Software	N/a
Cell population abundance	N/a
Gating strategy	N/a

☐ Tick this box to confirm that a figure exemplifying the gating strategy is provided in the Supplementary Information.

Magnetic resonance imaging

Experimental design

Design type	Task-based, block design
Design specifications	Visual and verbal items were presented for 3s in blocks. One block contained 10 faces (five fearful) and 10 words (two emotionally averse) and was followed by crosshair fixation. There was a total of 10 blocks (i.e., 100 faces and 100 words).To introduce jitter and ensure random sampling, a different interstimulus interval (3s) was used to the 2.75s repetition time of acquisition
Behavioral performance measures	The experimental session started with the in-scanner task. Participants were asked to memorize items that would be visually presented in the scanner, and explicitly informed about the subsequent out-of-scanner recall test. To foster deeper levels of encoding, participants were told to make a subjective decision about the pleasantness of the presented material,using a button-box.<br><br>Forty minutes after scanning, participants performed the out-of-scanner recognition task. Items that were encoded in-scanner were presented again, in random order and at the same speed as displayed the first time. Faces and words were tested separately. The same 100 stimuli were intermixed with an additional 50 novel items (i.e., faces or words) as foils. Participants were instructed to indicate whether items were remembered, familiar, or novel using a button box. For the postoperative scanning at one and ten years, an identical task with different visual and verbal stimuli was performed by all subjects.<br><br>Activation patterns at encoding of stimuli that were successfully remembered were used for subsequent event-related connectivity analysis.

Acquisition

Imaging type(s)	Functional and structural
Field strength	3T
Sequence & imaging parameters	Preoperatively and at 3–12-month follow-up, participants were scanned on a 3T GE Signa Excite HDx MRI scanner, with a 20-channel head coil. An axial 3D T1-weighted sequence (FSPGR) was acquired. For the memory fMRI, T2*-weighted gradient echo planar images (EPI) were acquired using 36 contiguous oblique axial slices per volume, 24-cm field of view, 2.5 mm slice thickness (0.3 mm gap), 96 x 96 matrix interpolated to 128 x 128 during image reconstruction, 2.5 in-plane resolution, and 2.5 SENSE factor (TE/TR = 25/27500 ms).<br><br>At the 10-year follow-up, data was acquired on a 3T GE Discovery MR750, with a 32-channel head coil. An axial 3D T1-weighted sequence (FSPGR) was acquired. Memory fMRI gradient-echo planar T2*-weighted fMRI was acquired using 50 contiguous oblique axial slices, 24-cm field of view, 2.4 mm slice thickness (0.1 mm gap), 64 × 64 matrix, 3.75 in-plane resolution, and 2.0 SENSE factor (TE/TR = 22/27500 ms).
Area of acquisition	Whole-brain scans with the field of view covering the temporal and frontal lobes, and slices were aligned on the sagittal view with the long axis of the hippocampus.

Diffusion MRI ☐ Used ☒ Not used

Preprocessing

Preprocessing software	Advanced Normalization Tools, Statistical Parametric Mapping (SPM) 12, Freesurfer.
Normalization	For both 3–12-month and 10-year timepoints, the anatomical 3D-T1 scan was registered to a scanner-specific template in MNI space. Short- and long-term functional imaging time-series were realigned to the mean image and time-corrected using SPM12. Normalization into standard anatomical space was done using EasyReg, a deep-leaning registration method accessible via Freesurfer.
Normalization template	The scanner-specific template was created from 30 healthy subjects, 15 individuals with left hippocampal sclerosis and 15 people with right hippocampal sclerosis, using high-resolution whole-brain EPI. (Sidhu et al., 2013. Brain)
Noise and artifact removal	The anatomical 3D-T1 scan underwent field bias correction with Advanced Normalization Tools. Functional imaging timeseries were normalized to the MNI-template using EasyReg which also performs EPI distortion correction (Iglesias. 2023. Scientific Reports).
Volume censoring	Imaging time-series were realigned to the mean image and time-corrected using SPM12.

Statistical modeling & inference

Model type and settings	Univariate fMRI analysis: general linear models.

Event-related spmT maps of subsequent memory effects were generated for each subject and separately for words or faces on SPM12 via random-effects analysis of a blocked design general linear model (GLM). Six regressors of interest were created; words and faces subsequently remembered, familiar, or forgotten. Six motion parameters were added as confounds. Resulting event-related statistical maps were used for subsequent single-level connectivity analyses (gPPI).

For verbal and visual memory separately, the subject-level gPPI model included three regressors: time-course of each event-related task-condition, timeseries of one MTL seed, and of the PPI term (task*seed interaction).

Resulting single-level gPPI t-contrasts of successful subsequent memory were used for group-level random-effects analyses.

Effect(s) tested

(1) Mixed ANOVAs, using flexible factorial design with IQ as confound regressor, were performed. One-sided t-contrasts were generated to look at differences in activations across scanning sessions between ATLR and control groups:
- 10-year connectivity > 3–12-month connectivity in left or right ATLR versus controls.
- 10-year connectivity < 3–12-month connectivity in left or right ATLR versus controls.

(2) Three-way ANCOVAs were conducted for each MTL seed and each successful memory contrast (words/faces), to investigate which differences in functional connectivity from 3–12-month and 10-year follow-ups were related with improvement in memory functions over this timeline. Positive correlations were examined using BMIPB (I or II) verbal and visual learning scores converted into age-normalized z-scores. Difference in z-scores between 3–12-month and 10-year follow-ups was used as continuous variables in three-ways ANCOVAs.

Specify type of analysis: ☒ Whole brain ☐ ROI-based ☐ Both

Statistic type for inference

(See Eklund et al. 2016)

Seed region to voxel-wise whole-brain analyses.

Correction

Given our a priori hypothesis of increased local medial temporal lobe (MTL) connectivity (including the fusiform gyrus), MTL connectivity was corrected for multiple comparisons at P < 0.05 voxel-wise, controlling for family-wise error rate via small volume correction.

Group comparison and correlation analyses generated highly specific MTL-to-whole-brain, longitudinal, event-related t-contrasts. Thus, at the extra-MTL level, functional connectivity is reported at an exploratory P < 0.001 threshold (uncorrected), alike previous longitudinal, event-related and network fMRI studies.

Models & analysis

n/a	Involved in the study
☐	☒ Functional and/or effective connectivity
☒	☐ Graph analysis
☒	☐ Multivariate modeling or predictive analysis

Functional and/or effective connectivity

Functional connectivity analysis: Psychophysiological interaction (PPI) analysis allows the investigation of task-modulated functional couplings between a seed and whole-brain regions. A generalized form of PPI was used to span the entire experimental space, wherein beta-estimates of all six event-related subsequent memory conditions were modelled. For each participant, ROI to voxelwise whole-brain analysis was performed based on a MATLAB (R2020b) script template from McLaren et al., that was adapted to work within the SPM12 framework.

Using each participant's event-related statistical map, each subject-level gPPI model included three regressors: the time-courses of event-related task conditions (subsequent verbal and visual memory), time-series of one MTL seed, and of the PPI term (i.e., task*seed interaction). All six event-related task conditions were modelled to better probe the specific effect of successful subsequent verbal and visual memory. Within each anatomical MTL seed, the average seed's time-course was extracted, over the eigen variable to avoid skewing the contribution of distinct voxels in different subjects.

The seed regressor identifies voxels that correlate with the MTL seed in general. Task*seed interaction term (i.e., PPI term) was formed by deconvolving the HRF from each MTL seed's time-course, multiplying the deconvolved output by each task condition's time-series separately, and finally re-convolving the time-series with the HRF. Deriving the PPI term from HRF deconvolution allows to model connectomics dynamics at the neural level. Subject-level resulting statistical parametric map presented whole-brain activations that were significantly more correlated with the seed during a specific event-related task than during the other conditions, based on the PPI term prediction and with physiologic and psychologic variables treated as nuisance regressors. For the study's scope, t-contrasts of areas functionally coupled to the MTL seed during encoding of either words or faces subsequently remembered were generated.
